# Mutational escape of CD8+ T cell epitopes: implications for prevention and therapy of persistent hepatitis virus infections

**DOI:** 10.1007/s00430-014-0372-z

**Published:** 2014-12-24

**Authors:** Joerg Timm, Christopher M. Walker

**Affiliations:** 1 Institute of Virology, University Hospital Düsseldorf, Heinrich-Heine-University, Universitätsstrasse 1, 40225 Düsseldorf, Germany; 2 Department of Pediatrics, The Ohio State University School of Medicine, Columbus, OH USA

**Keywords:** Hepatitis C virus, Hepatitis B virus, Immunotherapy, Exhaustion, PD-1

## Abstract

Over the past two decades, much has been learned about how human viruses evade T cell immunity to establish persistent infection. The lessons are particularly relevant to two hepatotropic viruses, HBV and HCV, that are very significant global public health problems. Although HCV and HBV are very different, the natural history of persistent infections with these viruses in humans shares some common features including failure of T cell immunity. During recent years, large sequence studies of HCV have characterized intra-host evolution as well as sequence diversity between hosts in great detail. Combined with studies of CD8+ T cell phenotype and function, it is now apparent that the T cell response shapes viral evolution. In turn, HCV sequence diversity influences the quality of the CD8+ T cell response and thus infection outcome. Here, we review published studies of CD8+ T cell selection pressure and mutational escape of the virus. Potential consequences for therapeutic strategies to restore T cell immunity against persistent human viruses, most notably HBV, are discussed.

## Introduction

Although the natural history of persistent HCV and HBV infections in humans is different, failure of T cell immunity is a common feature. Many of the same defects in T cell immunity have been described in both infections. For instance, an absence of sustained virus-specific CD4+ helper T cell immunity is central to development of chronic HBV and HCV infection. Cytotoxic CD8+ T cells persist in the liver after chronic infections are established, but lack key effector functions, a state defined as exhaustion. Whether exhaustion can be reversed by vaccination and/or interference with inhibitory signaling pathways that are active in exhausted CD8+ T cells is of current interest, especially for HBV where treatment options for long-term control of infection are limited. Some CD8+ T cell populations appear to be more exhausted than others during persistent virus infections, depending on whether viral class I epitopes have acquired mutations and evade recognition by CD8+ T cells. Substantial evidence supporting this concept has come from studies of HCV, a virus that is classified into seven different genotypes and multiple subtypes that differ up to 20 % at the amino acid level. During recent years, comparison of large number of HCV sequences has provided detailed insight into intra-host evolution as well as sequence diversity between hosts. Combined with studies of CD8+ T cell phenotype and function, it is now apparent that the response shapes viral evolution. In turn, HCV sequence diversity influences the quality of the CD8+ T cell response and thus infection outcome. Here, we review published studies of CD8+ T cell selection pressure and mutational escape of the virus.

## Evidence for CD8+ T cell selection pressure and HCV evolution

HCV particles harbor a positive-strand RNA genome that is directly translated in the cytosol of infected cells into a polyprotein approximately 3,000 amino acids in length. The polyprotein is cleaved by cellular and viral proteases into functional units. The virus encodes an RNA-dependent RNA polymerase (RdRp) located at the C-terminal end of the polyprotein (NS5B) that is needed for RNA-replication. Due to the lack of a proofreading function by the RdRp, viral replication is error prone and results in a swarm of multiple closely related but distinct viral variants termed as quasispecies. In response to selection pressure, viral variants that are best adapted for survival emerge from the quasispecies. It is now well established that CD8+ T cells are a significant source of selection pressure against HCV, most notably during the acute phase of infection. The CD8+ T cell response is highly individualized because multiple distinct allotypes with different epitope binding properties exist for HLA class I A, B and C genes. Transmission of the virus to a new host can therefore cause a dramatic shift in the epitope repertoire recognized by CD8+ T cells.

Evidence that HCV adapts to CD8+ T cell immune pressure by selection of substitutions in targeted epitopes was first obtained in the chimpanzee model [1, 2], which was well suited for these studies because viruses with a defined genome sequence could be used to initiate infection. Similar studies were subsequently carried out in infected humans under circumstances where the genomic sequences of donor and recipient viruses were available for comparison [3, 4]. Analysis of virus evolution in the chimpanzees and humans revealed a much higher rate of non-synonymous mutation in class I epitopes when compared with other regions of the HCV genome not under selection pressure by CD8+ T cells. The most comprehensive human study was performed in a cohort of eight people who inject drugs (PWID) with acute HCV genotype 1a infection [5]. Approximately 50 % of the class I epitopes acquired non-synonymous substitutions that facilitated escape from CD8+ T cell recognition. In turn, about 50 % of the substitutions in the non-structural proteins were associated with a detectable CD8+ T cell response. Other studies of human subjects are consistent with these findings [6–9]. The impact of CD8+ T cells on virus evolution has also been examined by aligning HCV amino acid polymorphisms and patterns of HLA class I allele expression in large populations of HCV-infected patients. These analyses provided additional evidence, even in the absence of donor virus genome sequences, that escape mutation is a very common and partially predictable feature of HCV infections that persist [10–13].

An increased rate of non-synonymous mutation in class I epitopes versus flanking regions was predominantly observed during the acute phase in human [6] and chimpanzee [14, 15] infections that ultimately persisted. As an example, in chimpanzees infected with clonal HCV genomes, non-synonymous mutation in class I epitopes was highest during the first 8–12 months of infection [14], just before CD8+ T cell selection pressure is thought to gradually fail. Once chronic infection was established, the rate of non-synonymous mutation is not substantially different between class I epitopes and flanking regions of the HCV genome. This suggests that the capacity of CD8+ T cells to recognize escape variants within epitopes, or to broaden to new epitopes, is limited during the chronic phase of infection. Consistent with these observations, escape mutations in HCV epitopes that arise during the acute phase are usually stable through decades of chronic infection [9, 16, 17] and de novo responses against new epitopes are equally rare [18]. This is quite different from the situation in persistent HIV infection where expansion of “escape-specific” CD8 T cells after selection of substitutions inside epitopes has been described [19, 20], most commonly for class I alleles such as HLA-B*27 and HLA-B*57 associated with protection from disease progression [21–24]. The HIV-specific CD8+ T cell response can also further broaden over the course of infection [25, 26]. Why the HCV-specific CD8+ T cell response fails to keep pace with virus evolution or broaden to new epitopes is not known. It might be explained by the concept of original antigenic sin, defined as impairment of a secondary immune response against a distinct but closely related antigen. This concept was described for CD8+ T cells in some murine models of virus infection [27], although it appears not to be universally operational in all settings [28]. Failure of CD8+ T cells to recognize new or escaped HCV epitopes may coincide with the profound, but as yet unexplained absence of HCV-specific CD4+ T cells during chronic infection [29]. Loss of CD4+ T cell help during the acute phase of infection might facilitate the initial emergence of escape variants. In support of this concept, one study in chimpanzees documented that antibody-mediated depletion of CD4+ T cells facilitated epitope escape from CD8+ T cells [30]. Further research is needed, however, to determine how an absence of helper activity during the acute and chronic phases of infection contributes to virus evolution and the capacity of CD8+ T cells to exert selection pressure.

## Functional mechanisms underpinning escape of class I epitopes

Mutation of even a single amino acid within an HCV epitope can defeat CD8+ T cell recognition through multiple mechanisms. One dominant mechanism involves mutation of an epitope anchor residue required for binding to the MHC class I molecule. Suboptimal binding leads to an unstable MHC class I/peptide complex and loss of the epitope. This appears to be the most frequent mechanism of immune escape for HIV because the majority of selected substitutions are located at these important anchor residues [31, 32]. Whether anchor residue substitutions are also the most common mechanism for mutational escape of HCV has not yet been determined. Amino acid substitutions at non-anchor residues can also impair CD8+ T cell recognition if they are located at the interface of the T cell receptor (TcR) and MHC class I/peptide complex. In this case, the variant epitope is still presented by the MHC class I molecule, but TcR binding is impaired. In the chimpanzee model, there is evidence that this mechanism of mutational immune escape is associated with a narrow TcR repertoire, whereas a broader repertoire may prevent viral escape, possibly due to enhanced cross-reactivity with more sequence variants of the epitope [33]. This observation suggests that CD8+ T cell populations may be qualitatively different in their capacity of immune selection. The ability to recognize multiple variants of a class I epitope may be the most important during the acute phase of infection before effective CD4+ and CD8+ T cell immunity is lost.

Finally, selected amino acid substitutions may alter processing of CD8+ T cell epitopes. Antigen presentation is a multi-step process that requires proteasomal degradation of viral proteins, transport to the endoplasmic reticulum, N-terminal trimming by amino peptidases and transport of the stabilized MHC class I/peptide complex to the cell surface. Many of these steps are sequence dependent and variation inside or near the epitope could alter this process. For example, it has been shown that substitutions inside the epitope can disrupt the endogenously processed epitope by preferential cleavage inside the epitope by the proteasome [34]. Altered epitope processing was also associated with substitutions in the C-terminal flanking region of an HLA-A*02-restricted epitope [35]. Whether such substitutions in the epitope flanking region are indeed selected by CD8+ T cell immune pressure is less clear. We recently identified a substitution five amino acids upstream of an HLA-B*51-restricted epitope in NS3 that is significantly more frequent in HLA-B*51-positive patients infected with HCV genotype 1a (unpublished). Functional analysis revealed that this substitution impaired antigen processing supporting that selection of altered epitope processing contributes to CD8+ T cell immune escape, although the overall extent of this viral escape pathway is unclear in hepatitis C.

## Relevance of CD8+ T cell escape mutations to infection outcome

Mutational escape of HIV from dominant CD8+ T cell responses is associated with increased replication of the persistent virus and progression to AIDS [36–38]. Whether escape mutations in class I epitopes determine whether acute HCV infection will ultimately resolve or persist is less clear. Viral mutational escape may simply be a consequence of continuous viral replication in the presence of selection pressure and not causal for viral persistence. Studies in chimpanzees experimentally infected with well-defined strains of the HCV revealed that class I escape mutations are much less common in infections that resolve spontaneously when compared with those that persist [2]. These studies are much more difficult to perform in humans because the acute phase of infection is often unrecognized due to mild or inapparent symptoms and the sequence of the donor virus is often not known. One study has nonetheless documented that escape mutations are also less frequent in acute resolving versus persistent HCV infections in humans [5]. Despite these associations, a convincing causal relationship between class I epitope escape and infection outcome has not been established. Such an analysis is likely to be complicated by multiple factors including the breadth of the CD8+ T cell response, whether viral fitness for replication is impaired by mutation of dominant epitopes, and the timing of CD4+ T cell loss and CD8+ T cell exhaustion during acute hepatitis C.

The impact of escape mutations and infection outcome is also likely to be influenced by HLA haplotype. Protective HLA class I alleles that correlate with better infection outcome have been identified for HIV [39] and HCV [40–42]. In HIV, there is evidence that immune control can be maintained despite selection of escape mutations in key epitopes presented by protective class I alleles like HLA-B*27 and HLA-B*57 [23, 43–45]. These key epitopes are constrained from mutation because of excessive fitness costs and selection of unfit viral variants may even be causal for viral containment [31, 46–48]. This would suggest that viral constraints determine the protective effect of particular HLA class I alleles. On the other hand, it has been argued that the epitopes presented by protective HLA class I alleles are targeted by T cells with stronger or more sustained antiviral activity [49]. There are strong arguments for both concepts, and successful control of infection might require a combination of both the “right” T cells against the “right” epitopes. It is notable that HLA-B*27 and HLA-B*57 are also associated with protection against HCV persistence. These two alleles, along with the protective allele HLA-A*03, present HCV epitopes that are nonetheless prone to mutational escape [50–52]. An important difference between these “protective” epitopes and “conventional” epitopes presented by other alleles may again be the genetic barrier to resistance. Mutational escape of HCV is also associated with varying degrees of fitness cost [13, 16, 17, 53]. Consequently, many escape mutations revert back to the prototype sequence in the absence of immune pressure [3, 54]. This has most recently been described in chronically infected pregnant women, where selection pressure is thought to be attenuated because of changes in maternal immunity to protect the fetus [55]. The genetic barrier to viral escape may be particularly high when the impact of mutation on virus replication is severe and/or a complex pattern of multiple substitutions is required to achieve full immune escape, as shown for dominant HCV epitopes presented by protective alleles [51, 56–58]. For immune escape from the dominant HLA-A*03- and HLA-B*57-restricted CD8+ T cell epitopes, secondary mutations were required to compensate for fitness costs associated with the primary escape mutation [51, 56, 58]. Interestingly, the dominant HLA-B*27 epitope was unique in that fitness was not impaired by mutational escape; here, cross-reactivity of epitope-specific CD8 T cells with different variants required multiple substitutions to achieve full escape [57].

Collectively, these data suggest that important qualitative differences exist between CD8+ T cell responses. For successful immune control and containment of HCV replication, a strong and ideally broadly cross-reactive CD8+ T cell response associated with a high genetic barrier to mutational immune escape is needed. Identification of these epitopes requires a combined analysis of the CD8+ T cell immune response in concert with viral sequences.

## Immunogenicity of CD8+ T cell escape variants

The clinical consequences of transmission of escape mutations are largely unclear for HCV. There are studies suggesting that the beneficial effect of particular HLA class I alleles is specific to individual HCV genotypes or even subtypes [52, 59]. This may be attributable to sequence differences in immunodominant CD8+ T cell epitopes restricted by these “protective” HLA alleles. For example, the advantageous effect of HLA-B*27 was so far only reported in HCV genotype 1 cohorts and was linked to a single epitope located in NS5B. Of note, the typically selected escape variant in genotype 1 resembles the prototype sequence in genotype 3. Interestingly, there is evidence that HLA-B*27 is not protective against HCV genotype 3a, suggesting that preexisting substitutions in the prototype HLA-B*27-restricted epitope precluded a dominant immune response [50]. Similarly, HLA-B*57 was reported to be associated with beneficial outcome only in cohorts infected predominantly with HCV genotype 1a [41, 52], whereas HLA-B*57 was not protective in cohorts infected with genotype 1b [40]. Again, it has been highlighted that genotype 1b typically harbors a variant of the dominant prototype epitope sequence in HCV genotype 1a that is associated with lack of the immune response [52]. Although these studies describe the impact of genotype or subtype-specific sequence differences on the outcome of infection, similar effects seem possible when it comes to transmission of escape mutations. Studies that directly address this are difficult to perform, because the exact viral sequence at the time of transmission is usually unknown. However, there are two large single-source outbreaks of hepatitis C for which the viral sequence of the inoculum is known and thus allow conclusions on the relevance of transmitted sequence variants in important CD8 T cell epitopes [9, 54]. In a large outbreak of HCV genotype 1b in Ireland, HLA-A*03 and HLA-B*27 were associated with spontaneous immune control, whereas HLA-B*08 was associated with viral persistence [40]. Interestingly, in a second very similar HCV genotype 1b outbreak in East Germany via the same transmission route, all three HLA alleles were neutral for the infection outcome [60]. This correlated with preexisting substitutions in the dominant epitopes presented by HLA-A*03 and HLA-B*27 in the source of the East-German outbreak, whereas the dominant epitope presented by HLA-B*08 was prototype. Notably, the same HLA-B*08 epitope carried substitutions in the source of the Irish outbreak possibly explaining the disadvantageous effect of HLA-B*08 specific for this cohort [54]. Although not conclusive, this strongly suggests that transmission of CD8 T cell escape variants is disadvantageous for the clinical outcome of a recipient who carries the same HLA alleles. Mutational escape of class I epitopes may also be an issue in the unique setting of pregnancy, where as noted above, some escape mutations can revert to wild-type sequence and cause an increase in viral fitness for replication [55]. It appears that the more fit viruses are transmitted vertically to infants, but more study is required to define the influence of maternal and paternal HLA haplotype on virus evolution and replication in this special population.

CD8 T cell responses against variant epitopes after mutational escape have only been reported in individual cases [16, 17]. It is unclear whether T cells with the relevant TCR are missing, whether they are anergic once chronic infection was established in the liver, or whether they are activated, but below the detection limit in the peripheral blood. Lack of T cells reactive with an escape variant of an HLA-A*02-restricted CD8 T cell epitope in NS3 was demonstrated [61]. In a study that analyzed CD8 T cell cross-reactivity between HCV genotype 1 and 3, an HLA-B*13-restricted epitope was identified that differed in one amino acid position [62]. Interestingly, in a cohort of people who inject drugs frequently exposed to genotype 1 and 3, co-existence of two distinct CD8 T cell populations against both genotypes, each without cross-reactivity, was reproducibly observed [62]. Although here the sequence differences were not the consequence of mutational escape, this suggests that a second CD8 T cell response against a closely related sequence variant can be activated also in HCV. Further studies are needed to address, whether and to what extent priming and activation of CD8 T cells directed against escape variants of HCV epitopes is possible during chronic infection.

## Impact of mutational escape on CD8+ T cell function and capacity for immune restoration

**Fig. 1:**
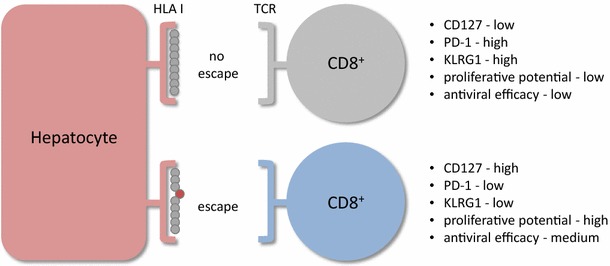
Impact of mutational antigen escape on the phenotype of specific CD8+ T cells during chronic HCV infection. Continuous antigen stimulation of CD8+ T cells results in T cell exhaustion associated with poor antiviral function. In contrast, after mutational escape CD8+ T cells develop a “memory” phenotype (CD127 high) with high proliferative potential upon stimulation with the cognate antigen and better antiviral efficacy

The number of epitopes that acquire escape mutations is variable, but 50 % or more do remain intact through the chronic phase of infection even though cognate CD8+ T cells survive in the liver. Exhaustion, characterized by a progressive loss of effector functions when continuously stimulated with antigen, almost certainly accounts for failure of these CD8+ T cells to control HCV replication [63]. Exhaustion is associated with increased expression of multiple inhibitory receptors such as PD-1, Tim-3, CTLA-4, 2B4 and LAG-3 and reduced expression of CD127 and the IL7 receptor-alpha chain that is required for self-renewal of memory populations [64–70]. Interestingly, this exhausted phenotype changes when escape mutations are introduced into class I-restricted epitopes (Fig. 1). HCV-specific CD8+ T cells targeting escaped epitopes display less PD-1 and more CD127 [64, 71, 72]. Moreover, proliferation is more robust upon antigen stimulation and they appear to retain or regain effector functions [64] when compared with CD8+ T cells with an exhausted phenotype that target intact epitopes. CD8+ T cells with a less exhausted phenotype also have an enhanced capacity to suppress HCV replication in a cell co-culture model after stimulation with a variety of cytokines or blockade of the PD-1 signaling pathway [73].

These observations suggest that immunotherapeutic vaccination or blockade of co-inhibitory signaling pathways may be more likely to reverse exhaustion of CD8+ T cells that target escaped versus intact class I epitopes. Studies in humans and chimpanzees suggest that both approaches can mobilize HCV-specific T cells and reduce virus load in a subset of chronically infected patients. For instance, reduction of virus load in a subset of patients after vaccination with a poxvirus vaccine that expressed non-structural HCV proteins has been reported [74]. Antibody-mediated blockade of the PD-1 co-inhibitory signaling pathway was also undertaken in humans [75] and chimpanzees [76], but again reduction of virus load was achieved only in a small subset of those who were treated. Of ten humans treated with a single high dose of anti-PD-1 antibodies (10 mg/kg BW), three had a remarkable reduction in HCV RNA in plasma of at least 10,000-fold which remained persistently undetectable in one of them after treatment. Of note, one patient with grade 4 ALT elevation (>10× upper limit of normal range) also had a significant decline in viral load. One chimpanzee of three treated with 5 weekly doses of anti-PD-1 antibodies (3 mg/kg BW) showed a substantial reduction in viremia that reverted to baseline levels with discontinuation of therapy [76]. The responder showed robust post-treatment expansion of intrahepatic HCV-specific CD4+ and CD8+ T cells when compared with the two non-responder animals [76]. Better characterization of the T cell response and viral sequences from the treated patients will be required to determine why many of those treated with the therapeutic poxvirus vaccine or anti-PD-1 antibodies failed to respond. Whether they had a more exhausted phenotype or primarily targeted epitopes that had acquired escape mutations remains to be determined. Although even after mutational escape of the epitope CD8 T cells may partially contribute to viral containment [55], it seems unlikely that activation and expansion of these CD8+ T cells is sufficient for viral eradication when the targeted antigen is not intact.

## Do escape mutations matter for prevention or treatment of chronic infection?

With the rapid development of type I interferon-free direct-acting antiviral agents against HCV, immunotherapeutic vaccination and/or blockade of co-inhibitory signaling pathways are unlikely to be considered for treatment of chronic hepatitis C. However, there is consensus that a prophylactic vaccine will be needed to prevent HCV infection in groups at high risk of infection [77, 78], and perhaps as importantly to prevent reinfection after expensive DAA-mediated cure of chronic hepatitis C [79]. One study in a chimpanzee cured of chronic infection with DAA revealed a substantial difference in responsiveness of T cells to escaped and intact epitopes upon reinfection with HCV 2 years later [80]. Only CD8+ T cells targeting epitopes that had escaped during the first infection expanded in liver upon reinfection. They provided transient control of virus replication that ultimately failed as viruses with new escape mutations in the dominant epitopes rapidly emerged. These observations, although very preliminary and restricted to a single treated individual, suggest that escape mutations can skew T cell responsiveness long after cure of infection. Vaccination after cure may be required to either restore CD8+ T cell responses to epitopes that were intact or to broaden the repertoire to new epitopes.

For HBV, direct-acting antivirals only suppress replication of the viral genome, usually without a significant impact on production of viral proteins thought to be responsible for T cell exhaustion. There is renewed interest in immunotherapy to reverse the immune tolerant state and reduce the risk of serious long-term liver diseases. Combination therapies to reduce production of viral antigens that exhaust T cells, and at the same time restore their function by vaccination or blockade of co-inhibitory signaling, are receiving attention. The promise of combination therapy using these approaches was elegantly demonstrated by Roggendorf and colleagues in the woodchuck model of persistent HBV infection [81, 82]. These findings almost certainly herald the start of a new era of therapy for chronic hepatitis B, a disease that affects more than 240 million people globally. Importantly, escape mutations in class I epitopes have been described in patients with chronic hepatitis B [83], but whether they are important for CD8+ T cell evasion or alter the course of infection is very poorly understood when compared with HIV and HCV infection. Translation of very promising new therapies from animals to humans would likely benefit from a more detailed understanding of how escape mutations influence the repertoire, phenotype and function of CD8+ T cells in humans persistently infected with HBV.
